# Supplementary material for: Promising Prognosis Marker Candidates on the Status of Epithelial–Mesenchymal Transition and Glioma Stem Cells in Glioblastoma
Source: Cells. 2019 Oct 24;8(11):1312. doi: 10.3390/cells8111312 (PMC6912254; doi:10.3390/cells8111312)
Supplement: Supplementary file 1 [file cells-08-01312-s001.pdf]

Table S1

Table S1. Clinical data of patients with glioblastoma examined in the study.

	Training data set TCGA (n = 151)	Validation data set CGGA (n = 135)
Gender (n)		
Female	55	51
Male	96	84
Age (years old)		
Median	61.7	46.5
Average	60.7	48
Min	21.7	8
Max	89.3	79
Overall survival (months)		
Median	9.3	12.3
Average	11.4	20.7
Min	0.2	0.6
Max	54	126.7
IDH mutation (n)		
Wild type	141	95
Mutation	7	40
Unknown	3	-
MGMT methylation (n)		
Methylated	54	-
Unmethylated	66	-
Unknown	31	-

Table S2

Table S2. List of the genes related epithelial-mesenchymal transition and glioma stem cell markers.

Epithelial markers	Mesenchymal markers	Glioma markers	Glioma stem cell markers	Molecular target therapy	Potential glioma biomarkers
CDH1	ACTA2	ATRX	BMI1	COX2	AHSG
CLDN1	CDH11	BRAF	CD44	DEPTOR	CD63
COL4A1	CDH2	CDK4	FUT4	EGFR	CHI3L1
DSC1	COL1A1	CDKN2A	GFAP	FLT1	FABP5
DSG3	COL3A1	CHI3L1	ITGA6	FLT4	FOXM1
DSP	CTNNB1	COX2	KLF4	HDAC1	FSTL1
KRT1	ETS1	DEPTOR	L1CAM	HDAC10	GADD45A
KRT18	FN1	EGFR	MSI1	HDAC11	GFAP
LAMA1	FOXC2	FLT1	MYC	HDAC2	GSN
MUC1	GSC	FLT4	NANOG	HDAC3	IDH1
NID1	ITGA5	H3F3A	NES	HDAC4	IDH2
OCLN	ITGAV	HDAC1	OLIG2	HDAC5	IGFBP2
SDC1	ITGB1	HDAC10	POU5F1	HDAC6	IQGAP1
TJP1	ITGB6	HDAC11	PROM1	HDAC7	MGMT
	LAMA5	HDAC2	SALL4	HDAC8	MMP9
	LEF1	HDAC3	SOX2	HDAC9	NAMPT
	RUNX2	HDAC4	STAT3	KDR	PEA15
	S100A4	HDAC5		MLST8	PTPRZ1
	SNAI1	HDAC6		MTOR	S100A10
	SNAI2	HDAC7		PDGFRA	S100A6
	TWIST1	HDAC8		PGF	SPP1
	VIM	HDAC9		RICTOR	STARD13
	ZEB1	HIF1A		RPTOR	TBCA
		IDH1		SIRT1	TIMP4
		IDH2		SIRT2	
		KDR		SIRT3	
		MET		SIRT4	
		MGMT		SIRT5	
		MLST8		SIRT6	
		MTOR		SIRT7	
		NF1		VEGFA	
		PDGFRA		VEGFB	
		PGF		VEGFC	
		PIK3CA		VEGFD	
		PIK3CB			
		PIK3CG			
		PTEN			
		RB1			
		RICTOR			
		RPTOR			
		SIRT1			
		SIRT2			
		SIRT3			
		SIRT4			
		SIRT5			
		SIRT6			
		SIRT7			
		TERT			
		TP53			
		VEGFA			
		VEGFB			
		VEGFC			
		VEGFD			

Table S3

Table S3. Gene description of the 22 gene marker candidates.

Gene symbol	Description
BMI1	BMI1 proto-oncogene, polycomb ring finger(BMI1)
CDH1	cadherin 1(CDH1)
CLDN1	claudin 1(CLDN1)
DSG3	desmoglein 3(DSG3)
EGFR	epidermal growth factor receptor(EGFR)
FLT1	fms related tyrosine kinase 1(FLT1)
FN1	fibronectin 1(FN1)
GADD45A	growth arrest and DNA damage inducible alpha(GADD45A)
GFAP	glial fibrillary acidic protein(GFAP)
HDAC1	histone deacetylase 1(HDAC1)
HDAC3	histone deacetylase 3(HDAC3)
HDAC7	histone deacetylase 7(HDAC7)
IGFBP2	insulin like growth factor binding protein 2(IGFBP2)
L1CAM	L1 cell adhesion molecule(L1CAM)
MGMT	O-6-methylguanine-DNA methyltransferase(MGMT)
MMP9	matrix metallopeptidase 9(MMP9)
OLIG2	oligodendrocyte lineage transcription factor 2(OLIG2)
PGF	placental growth factor(PGF)
PTEN	phosphatase and tensin homolog(PTEN)
SIRT1	sirtuin 1(SIRT1)
STAT3	signal transducer and activator of transcription 3(STAT3)
TIMP4	TIMP metallopeptidase inhibitor 4(TIMP4)

Table S4

Table S4. Gene ontology of the 22 gene marker candidates.

GO biological process complete	P-value	Category
positive regulation of cell population proliferation (GO:0008284)	3.74E-07	BP
positive regulation of multicellular organismal process (GO:0051240)	2.72E-06	BP
system development (GO:0048731)	4.50E-06	BP
regulation of protein metabolic process (GO:0051246)	8.02E-06	BP
positive regulation of cellular process (GO:0048522)	1.16E-05	BP
developmental process (GO:0032502)	3.67E-05	BP
multicellular organism development (GO:0007275)	4.38E-05	BP
cell differentiation (GO:0030154)	4.81E-05	BP
cellular developmental process (GO:0048869)	7.16E-05	BP
regulation of cell migration (GO:0030334)	7.58E-05	BP
multicellular organismal process (GO:0032501)	8.74E-05	BP
histone H3 deacetylation (GO:0070932)	1.02E-04	BP
positive regulation of biological process (GO:0048518)	1.37E-04	BP
regulation of cell motility (GO:2000145)	1.44E-04	BP
anatomical structure development (GO:0048856)	1.64E-04	BP
negative regulation of cellular process (GO:0048523)	1.87E-04	BP
regulation of cell population proliferation (GO:0042127)	2.33E-04	BP
regulation of multicellular organismal development (GO:2000026)	2.78E-04	BP
regulation of locomotion (GO:0040012)	3.02E-04	BP
regulation of cellular component movement (GO:0051270)	3.32E-04	BP
positive regulation of cell migration (GO:0030335)	3.61E-04	BP
response to drug (GO:0042493)	4.08E-04	BP
positive regulation of cell motility (GO:2000147)	5.00E-04	BP
positive regulation of developmental process (GO:0051094)	5.11E-04	BP
positive regulation of cellular component movement (GO:0051272)	6.28E-04	BP
positive regulation of locomotion (GO:0040017)	7.83E-04	BP
negative regulation of biological process (GO:0048519)	1.26E-03	BP
positive regulation of protein modification process (GO:0031401)	2.38E-03	BP
histone deacetylation (GO:0016575)	2.54E-03	BP
positive regulation of cellular protein metabolic process (GO:0032270)	2.57E-03	BP
NAD-dependent histone deacetylase activity (GO:0017136)	1.16E-05	MF
NAD-dependent protein deacetylase activity (GO:0034979)	1.44E-05	MF
histone deacetylase activity (GO:0004407)	1.21E-04	MF
protein deacetylase activity (GO:0033558)	1.37E-04	MF
identical protein binding (GO:0042802)	2.67E-04	MF
deacetylase activity (GO:0019213)	6.71E-04	MF
histone deacetylase activity (H3-K14 specific) (GO:0031078)	8.99E-04	MF
NAD-dependent histone deacetylase activity (H3-K14 specific) (GO:0032041)	8.99E-04	MF
hydrolase activity, acting on carbon-nitrogen (but not peptide) bonds, in linear amides (GO:0016811)	4.51E-03	MF
protein domain specific binding (GO:0019904)	2.08E-02	MF
enzyme binding (GO:0019899)	2.26E-02	MF
kinase binding (GO:0019900)	3.08E-02	MF
hydrolase activity, acting on carbon-nitrogen (but not peptide) bonds (GO:0016810)	3.63E-02	MF
apical plasma membrane (GO:0016324)	1.69E-03	CC
apical part of cell (GO:0045177)	4.70E-03	CC

Note: GO; gene ontology, BP; biological process, MF; molecular function, CC; cellular component.

Figure S1

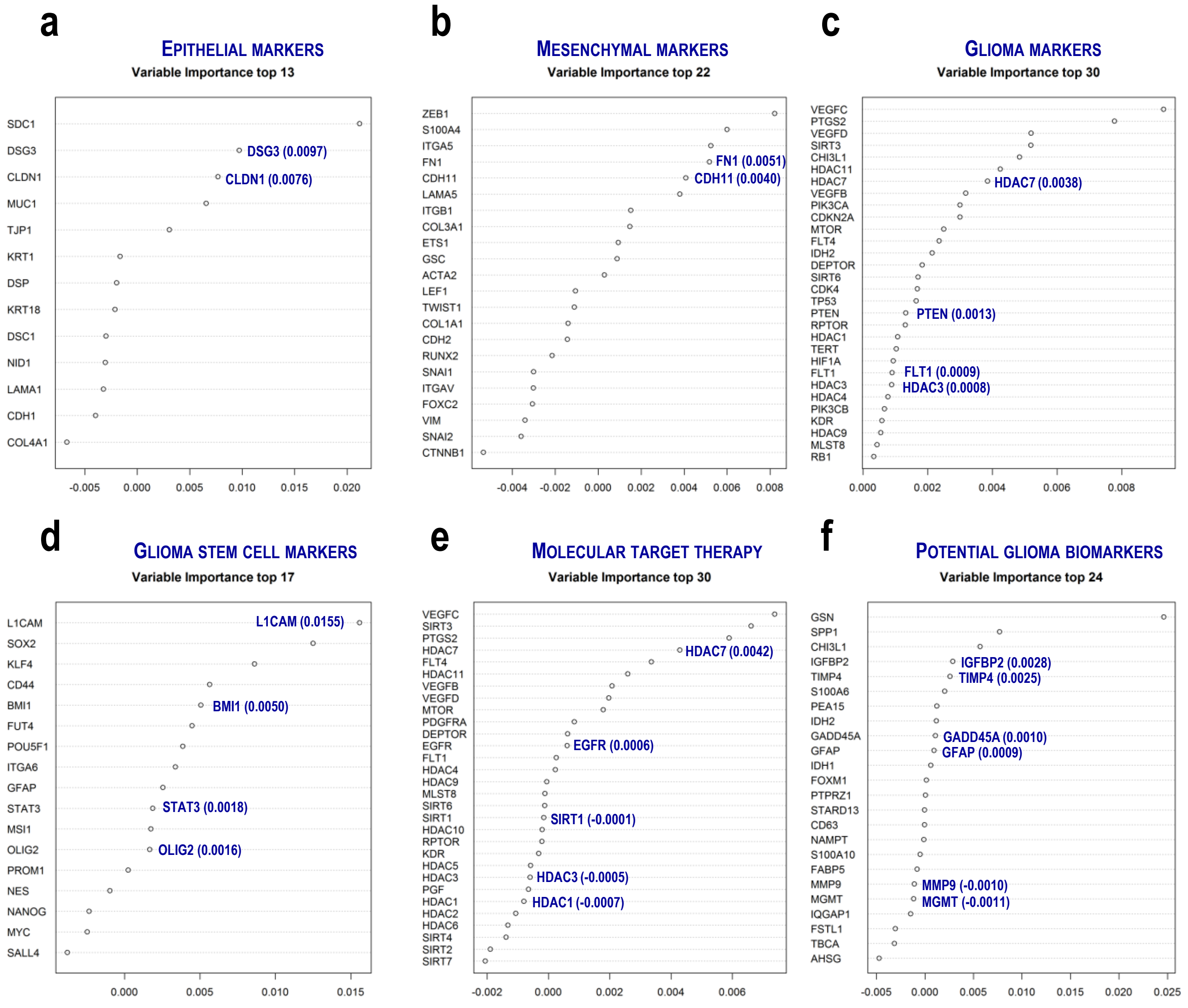

Figure S1. Variable importance factors in the analysis for epithelial-mesenchymal transition and glioma in 151 patients with glioblastoma multiforme. The variable importance measured with a Random Forests model are shown. The gene symbols in the panels represent significant genes with a Cox proportional hazards regression analysis (**a**) Epithelial marker gene set. (**b**) Mesenchymal marker gene set. (**c**) Glioma marker gene set. (**d**) Glioma stem cell marker gene set. (**e**) Molecular target therapy gene set. (**f**) Potential glioma marker gene set. The numbers in the parentheses in panels denote the values of variable importance factors.

Figure S2

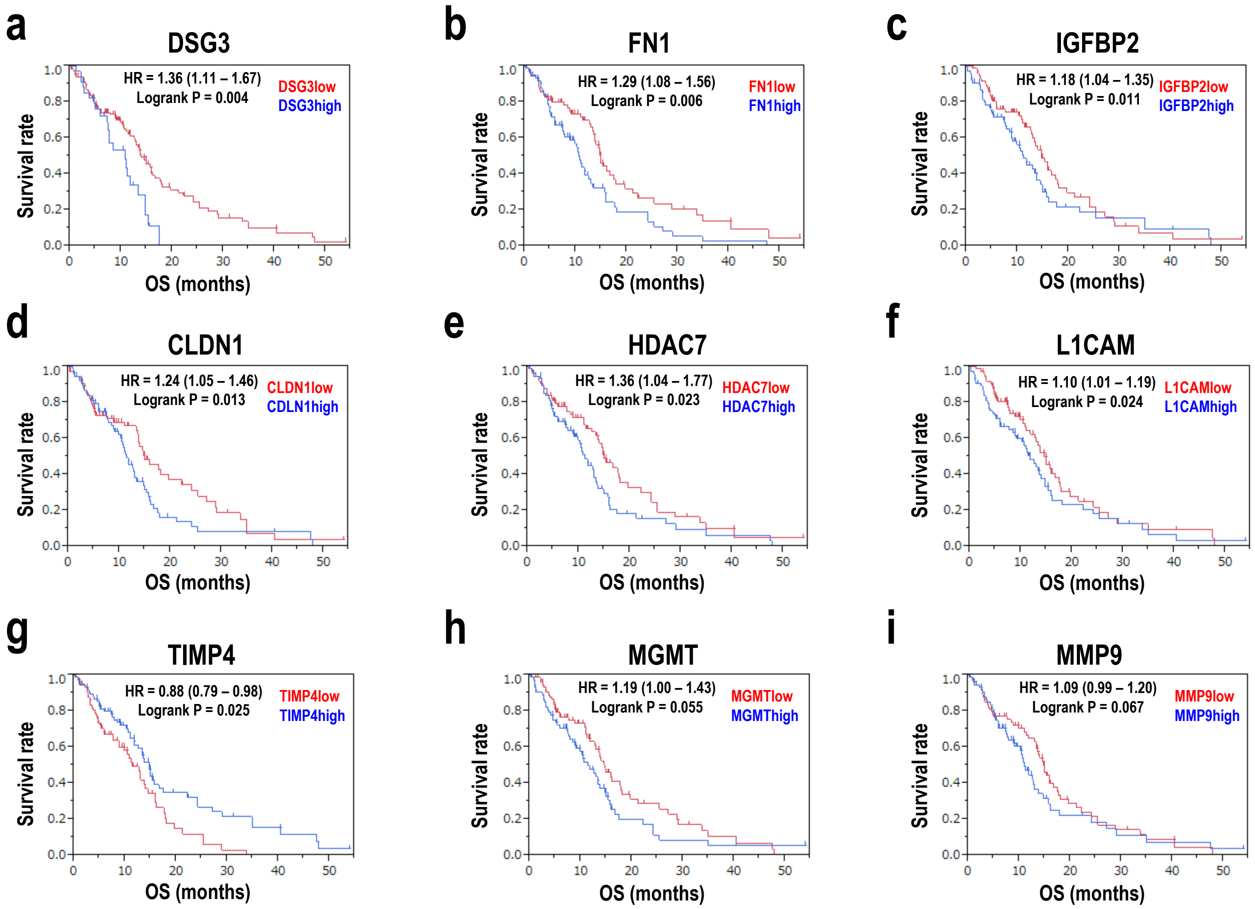

Figure S2. Survival distribution estimated using a single gene expression with Kaplan-Meier method. (**a**) DSG3. (**b**) FN1. (**c**) IGFBP2. (**d**) CLDN1. (**e**) HDAC7. (**f**) L1CAM. (**g**) TIMP4. (**h**) MGMT. (**i**) MMP9. HR; hazard ratio, OS; overall survival.

Figure S3

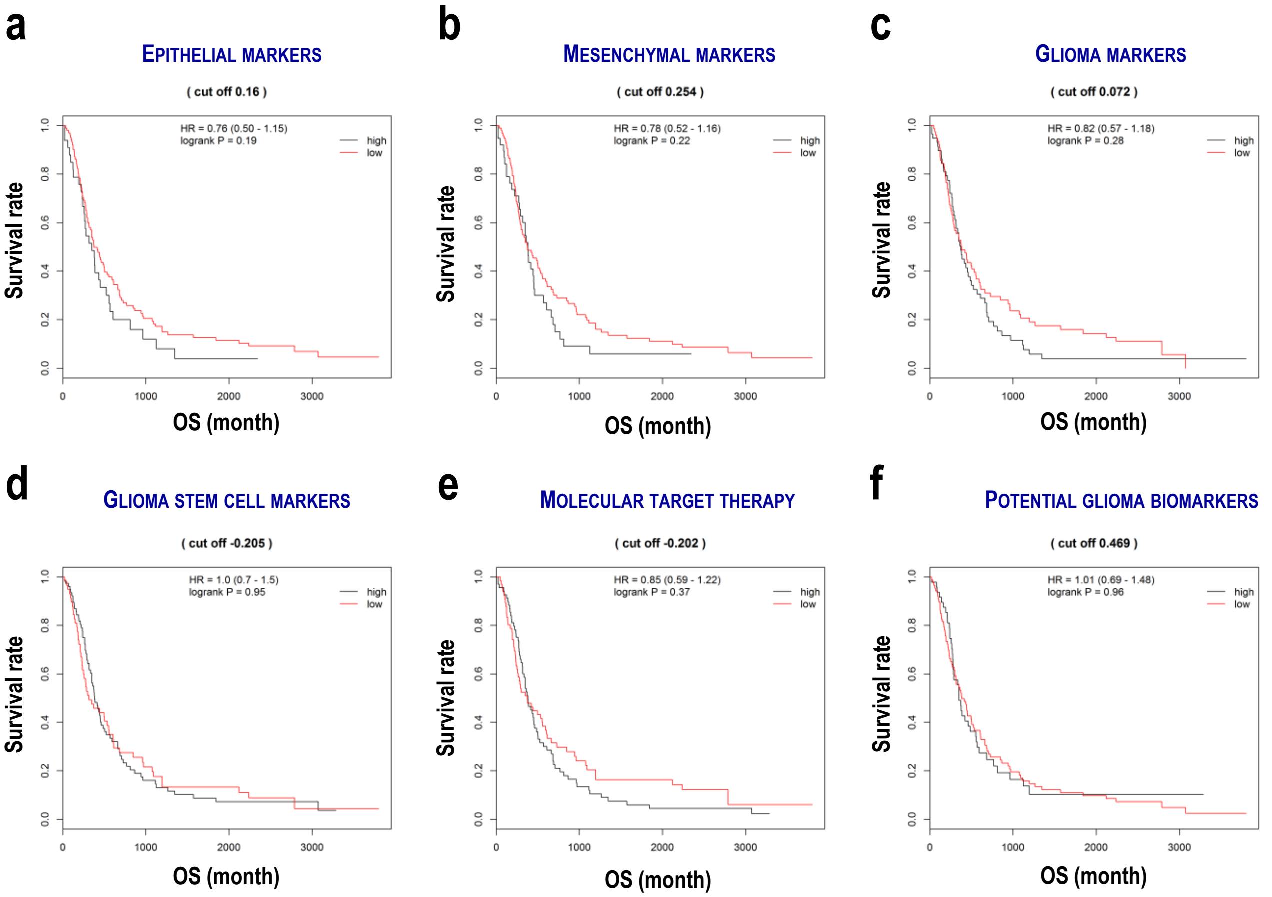

Figure S3. Survival distribution of the subgroups divided by the prognosis prediction formulas in the CGGA gene sets in glioblastoma multiforme. (**a**) Epithelial marker set. (**b**) Mesenchymal marker gene set. (**c**) Glioma marker gene set. (**d**) Glioma stem cell marker gene set. (**e**) Molecular target therapy gene set. (**f**) Potential glioma marker gene set. OS; overall survival, HR; hazard ratio, cut off score; a median score from a prognosis prediction formula. High and low denote the subgroups of the patients associated with the over and under median scores of the prognosis scores.

Figure S4

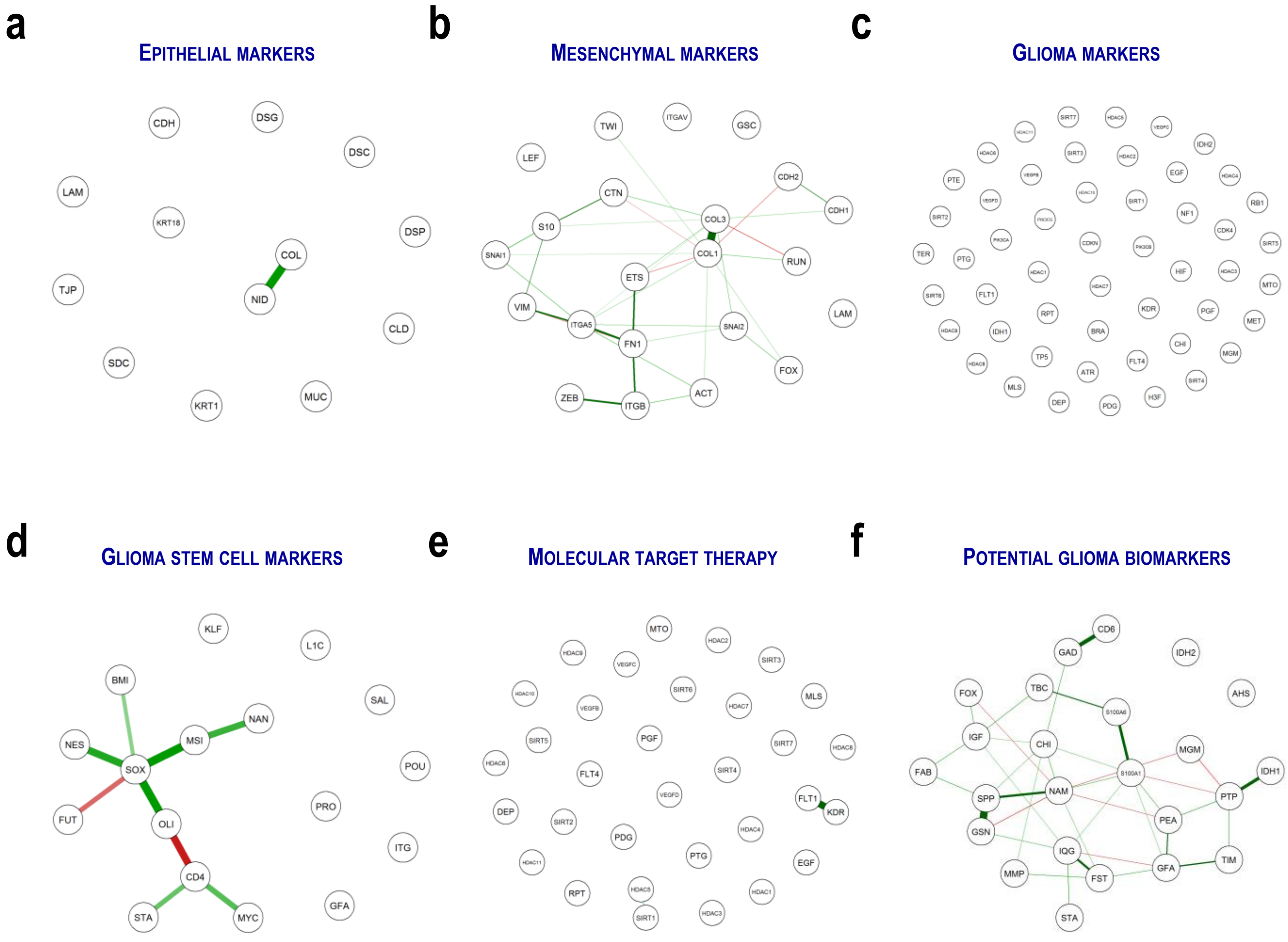

Figure S4. Genetic interaction networks based on the gene expression within the CGGA gene sets in glioblastoma multiforme. The graphical lasso estimation of the network models are drawn. (**a**) Epithelial marker gene set. (**b**) Mesenchymal marker gene set. (**c**) Glioma marker gene set. (**d**) Glioma stem cell marker gene set. (**e**) Molecular target therapy gene set. (**f**) Potential glioma marker gene set. The genes with significant hazard ratios are highlighted. Red and green represent poor and good prognosis, respectively.

Figure S5

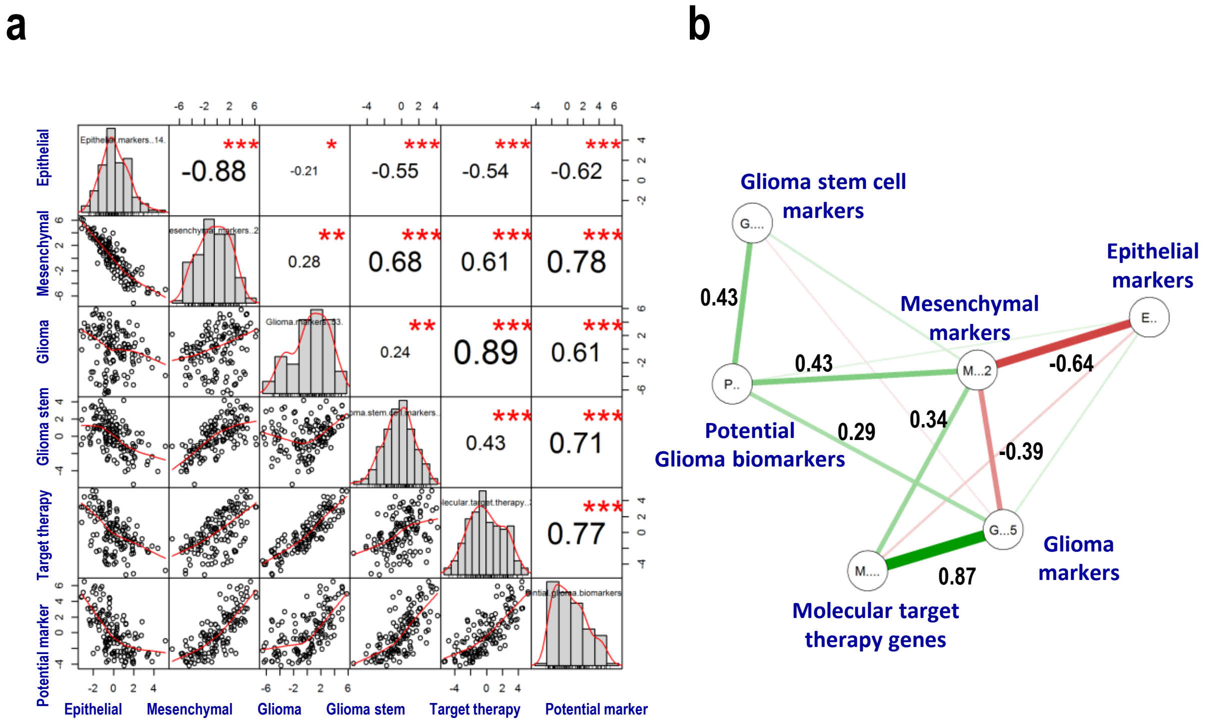

Figure S5. Correlation among the CGGA gene sets in glioblastoma multiforme. (**a**) Score correlation in glioblastoma multiforme. (**b**) The graphical lasso estimation with the network model of the gene sets related to epithelial-mesenchymal transition and glioma in glioblastoma multiforme.

Figure S6

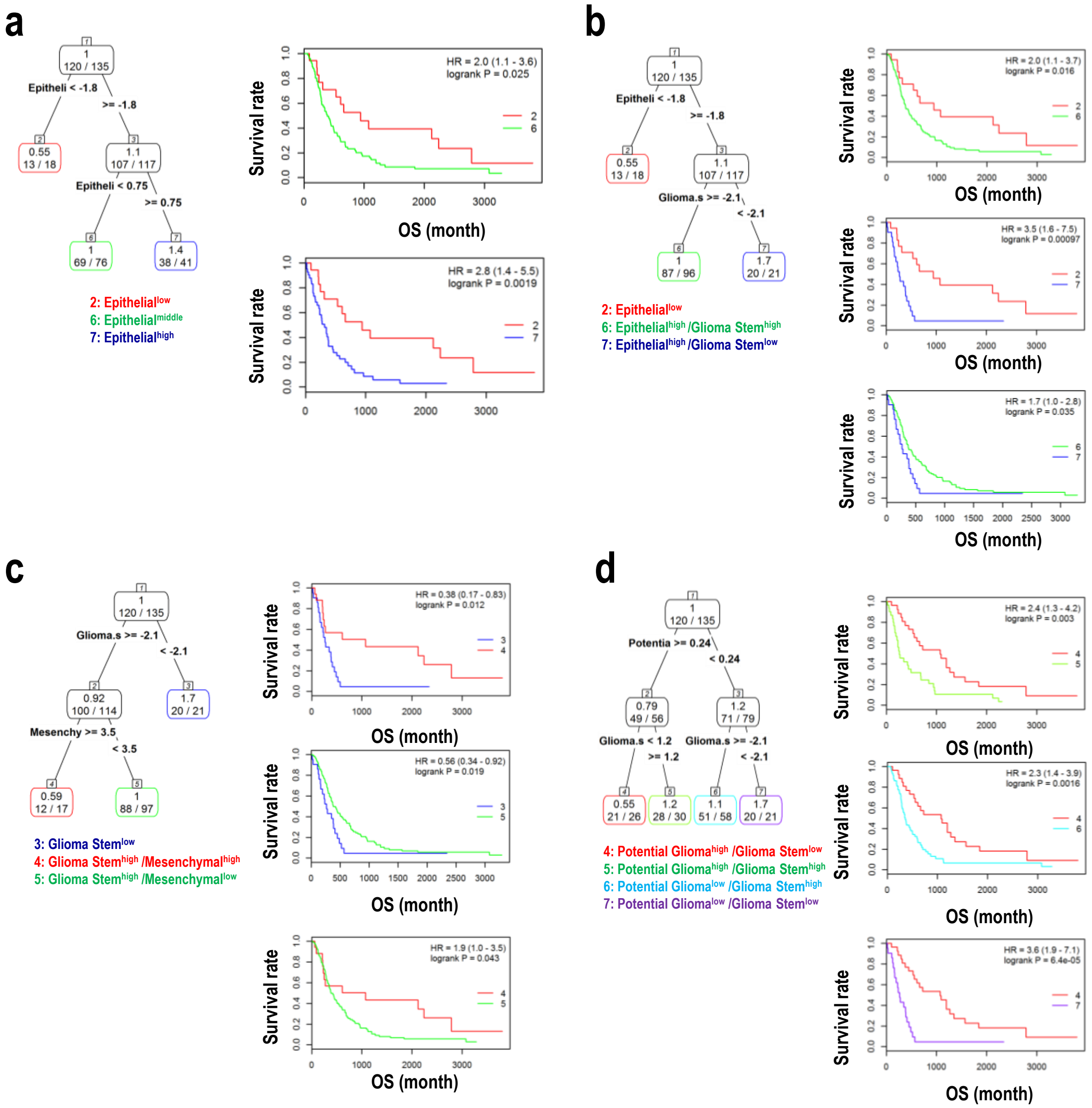

Figure S6. Survival tree analysis for the EMT statuses and glioma markers in the CGGA glioblastoma multiforme data set. (**a**) Epithelial status. (**b**) Epithelial status and glioma stem cell markers. (**c**) Glioma stem cell markers and mesenchymal status. (**d**) Potential glioma biomarkers and glioma stem cell markers. HR; hazard ratio, OS; overall survival.
